# Clinicopathologic Features and Management of Incidentally Detected Sub-centimeter Rectal Neuroendocrine Tumors: A Case Series

**DOI:** 10.7759/cureus.97779

**Published:** 2025-11-25

**Authors:** Taranjit Kaur, Harnoor G Galani, George Andriashvili, Nishan Dogra, Peter M Houston, Grant Williams

**Affiliations:** 1 Pathology, William Carey University College of Osteopathic Medicine, Hattiesburg, USA; 2 Basic Sciences, University of California, Riverside, Riverside, USA; 3 Basic Sciences, University of Southern California, Los Angeles, USA; 4 Anatomic Pathology/Clinical Pathology, 81st Medical Group at Keesler Air Force Base, Biloxi, USA; 5 Anatomic Pathology/Clinical Pathology/Cytopathology, 81st Medical Group at Keesler Air Force Base, Biloxi, USA

**Keywords:** endoscopic diagnosis, histopathology, minimally invasive resection, prognostic evaluation, rectal neuroendocrine tumor

## Abstract

Rectal neuroendocrine tumors (NETs) are rare but increasingly recognized due to widespread colonoscopy screening for colorectal cancer and other gastrointestinal conditions. Advances in endoscopic technology have led to earlier and more frequent detection of small, often asymptomatic rectal lesions. While rectal NETs generally exhibit an indolent course, accurate histologic grading and staging, including assessment of depth of invasion and lymphovascular or perineural invasion, remain critical for risk stratification and treatment selection. There is limited real-world data on incidentally detected (<1 cm) rectal NETs across mixed clinical indications.

We retrospectively analyzed the clinicopathologic characteristics and management of three patients diagnosed with small (<1 cm), grade 1 (G1) rectal NETs that were incidentally detected during endoscopic evaluations for various indications, including gastroesophageal reflux disease workup, ulcerative colitis surveillance, and routine colon cancer screening. All cases were reported using a standardized pathology protocol for colorectal NETs that included documentation of tumor size, depth of invasion, margin status, and lymphovascular invasion. All three patients exhibited well-differentiated G1 rectal NETs with Ki-67 ≤3% and minimal mitotic activity, confined to the submucosa and without lymphovascular or perineural invasion. Endoscopic resection was performed in all cases, with no complications or residual disease noted, and clinical follow-up ranging from 6 to 18 months showed no recurrences or disease progression. This case series highlights the importance of vigilant endoscopic recognition, standardized histopathological reporting, and risk-based management in identifying small rectal NETs at an early, highly manageable stage. Our findings align with current guideline-based recommendations that support conservative management with endoscopic resection and surveillance for most subcentimeter, well-differentiated rectal NETs, and add to the limited real-world evidence on incidentally detected lesions across diverse clinical contexts.

## Introduction

A small fraction of gastrointestinal tumors are rectal neuroendocrine tumors (NETs), also known as carcinoid tumors. Since the implementation of high-resolution endoscopic technologies, the detection of small, asymptomatic rectal NETs has significantly increased. It is anticipated that, due to the U.S. Preventive Services Task Force recently reducing the recommended age for colorectal cancer screening from 50 to 45 years, more cases will be identified [1]. Statistically, rectal NETs occur in about 1 out of every 100,000 individuals annually. Approximately 80 to 90 percent are less than 1 centimeter in size and remain confined to the submucosa at the time of diagnosis [2]. This change highlights the importance of identifying and treating these uncommon yet frequently occurring lesions, which are often discovered incidentally during evaluations for unrelated gastrointestinal concerns [3,4]. Histologically, rectal NETs are composed of uniform neuroendocrine cells that typically express markers such as synaptophysin and chromogranin and are graded using the Ki-67 proliferation index.

Although small rectal NETs typically grow slowly, precise histological staging and grading are still necessary to determine the best course of treatment and to evaluate the long-term prognosis [5-7]. In a 2021 retrospective study, the authors found that rectal NET lesions measuring 10 mm or less may still harbor nodal metastases in some cases, underscoring the importance of early detection [8]. The literature also presents strong evidence that chronic mucosal inflammation may play a role in the proliferation of neuroendocrine cells and the growth of tumors. Recognized connections exist between rectal NETs and chronic inflammatory diseases, such as ulcerative colitis (UC) [6,9-11]. There is limited real-world data on incidentally detected (<1 cm) small, rectal NETs across mixed indications. This case series explores the clinicopathologic features and clinical outcomes of three patients with small (<1 cm), Grade 1 rectal neuroendocrine tumors that were inadvertently discovered during routine endoscopic evaluations.

## Case presentation

All three tumors were reported using our institutional synoptic format based on the College of American Pathologists (CAP) protocol for colorectal NETs, including documentation of tumor size, depth of invasion, margin status, and lymphovascular and perineural invasion.

Patient A

**Figure 1:**
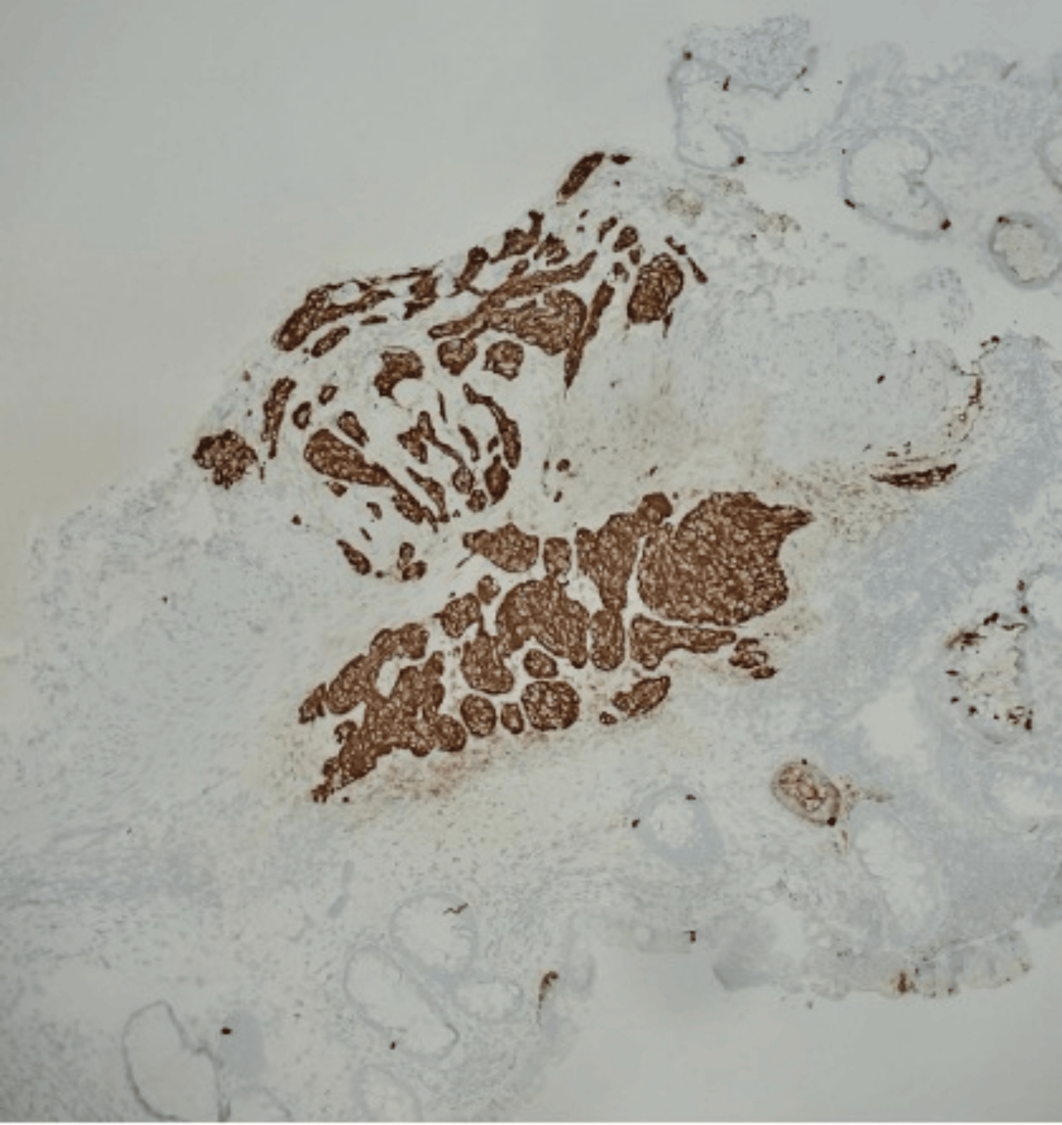
Low-power view of lesional cells expressing Lu-5.

**Figure 2:**
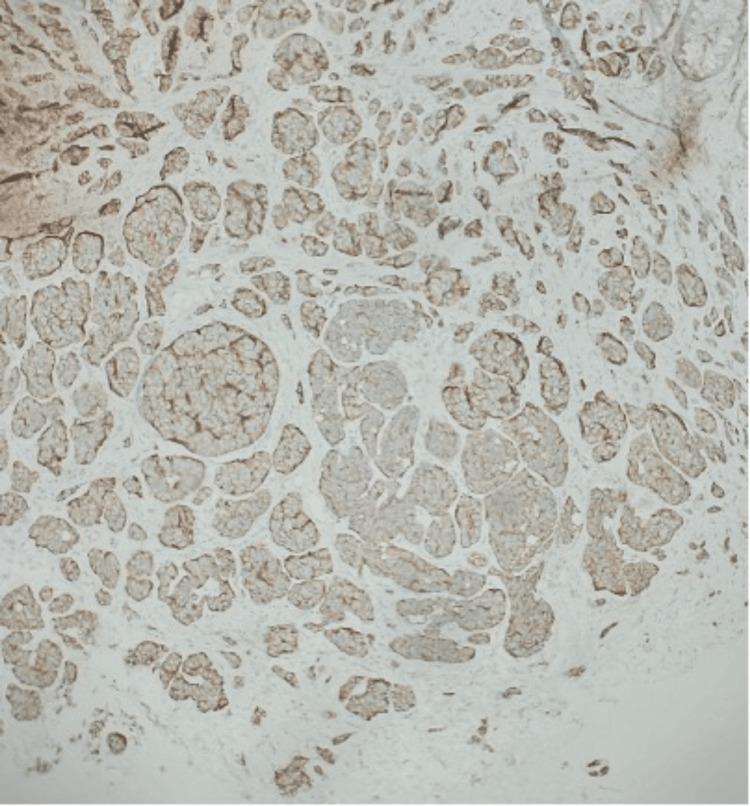
Low-power view of lesional cells expressing Synaptophysin.

A 70-year-old male patient with a medical history of gastroesophageal reflux disease and colonic polyps received both an endoscopy and a colonoscopy. During the procedures, biopsies of the stomach, esophagus, and rectum were collected for analysis, which revealed a benign fundic gland polyp and a rectal polyp. The rectal polyp revealed a lesion measuring 1.0 × 0.6 × 0.4 cm^3^. Pathology of the lesion showed a grade 1 (G1) well-differentiated NET with features of monotonous cells arranged in sheets, nests, and ribbons with no significant atypia. The tumor was evaluated with immunohistochemical stains and found to be positive for synaptophysin, Lu-5, and CDX-2 (Figures 1, 2). Chromogranin was negative. The Ki-67 proliferation index was approximately 2%, with one mitotic figure per 2 mm². Neither lymphovascular nor perineural invasion was observed, and the resection margins were negative. During follow-up, the tumor was subsequently managed with a complete endoscopic resection, which achieved complete removal with no residual disease.

Patient B

**Figure 3:**
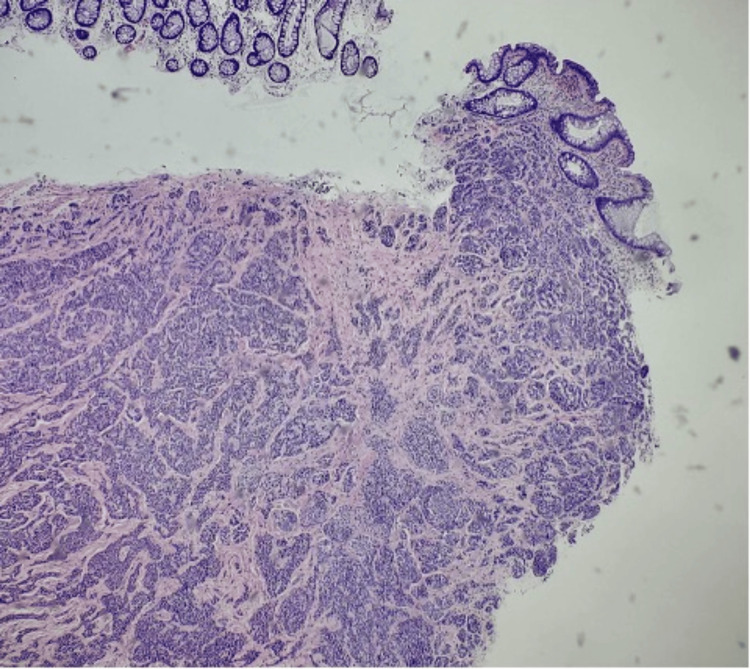
Low-power view of lesional cells showing infiltrative growth in a nested pattern.

**Figure 4:**
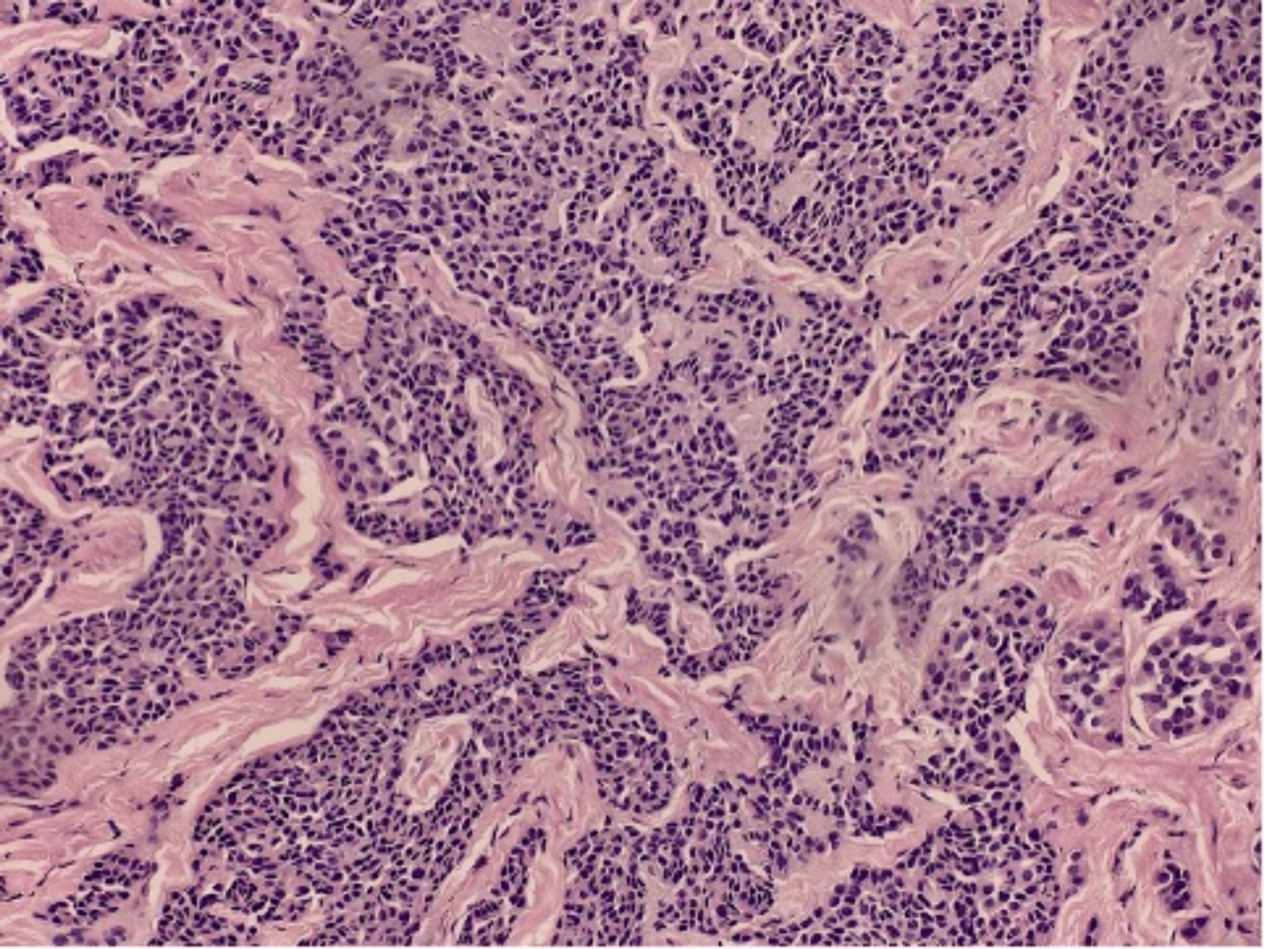
Medium-power view of infiltrative lesional cells in trabeculae.

A 38-year-old male patient with a history of gastroesophageal reflux disease with Helicobacter pylori underwent an endoscopic assessment to evaluate recent bright red blood per rectum. A 1.5 mm rectal lesion was found and removed, and the rectal polyp was sent for histological examination. Pathology revealed a G1 well-differentiated NET, which exhibited nests, rosettes of monotonous cells, and no discernible atypia (Figures 3, 4).

The tumor was evaluated with immunohistochemical stains and found to be positive for synaptophysin and Lu-5 and negative for chromogranin. The Ki-67 proliferation rate was 2.6%, and mitotic activity was less than one mitotic figure per 2 mm². The lesion was positioned at the tissue border and had a maximum size of 1.5 mm, indicating that complete excision may not have been achieved. Furthermore, no deeper infiltration, lymphovascular invasion, or perineural invasion was seen. During follow-up, the patient was managed clinically with surveillance and exhibited no symptoms of recurrence.

Patient C

**Figure 5:**
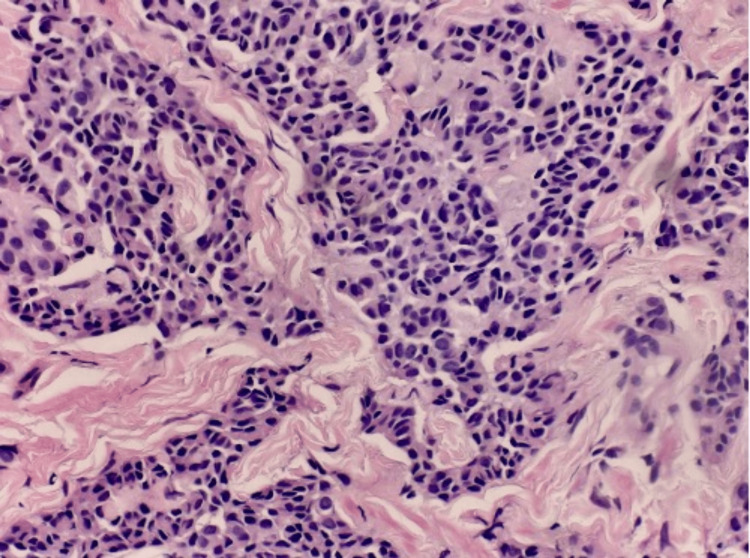
High-power view showing infiltrative lesional cells with neuroendocrine morphology.

**Figure 6:**
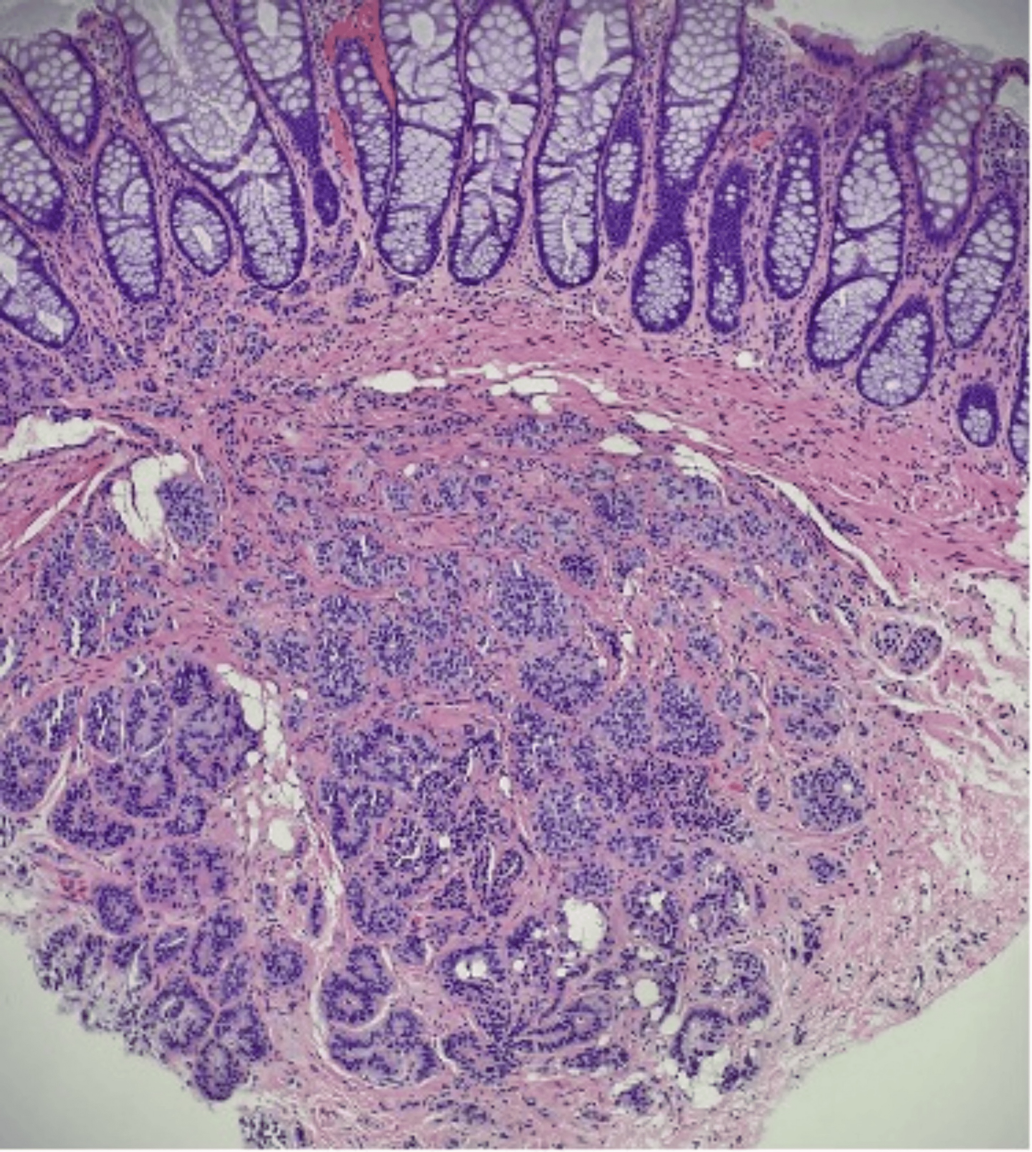
Low-power view showing lesional cells underlying normal colonic mucosa.

During routine disease surveillance, a 60-year-old male patient with a known medical history of UC underwent a colonoscopy. The rectum was one of several colonic segments from which biopsies were obtained. Pathology revealed a portion of a G1 well-differentiated NET with submucosal invasion found during the rectal biopsy (Figures 5, 6).

Synaptophysin and Chromogranin A expression were detected with immunohistochemical staining. The Ki-67 index was less than 2%. There was no evidence of unusual characteristics, lymphovascular invasion, or perineural invasion. The residual colon showed quiescent colitis with no dysplastic features. The rectal NET was conservatively handled with endoscopic excision. The patient remained asymptomatic during follow-up with no residual or recurrent disease. The clinical, histopathological, and immunohistochemical findings of all three patients are summarized in Table 1.

**Table 1:** Clinical and pathological characteristics of three patients with neuroendocrine tumors (NETs). Footnotes: IHC markers refer to immunohistochemical staining results used to characterize neuroendocrine tumors. SYN (Synaptophysin) and CHR (Chromogranin) are neuroendocrine markers. LU5 (Lu-5) is a Pan Cytokeratin marker, and CDX2 is an intestinal differentiation marker. Ki-67 represents the proliferation index and is already included in the histopathological features row. "Not Performed" indicates that the respective test was not conducted for that patient. GERD: Gastroesophageal reflux disease

Parameter	Patient A	Patient B	Patient C
Age/Sex	70-year-old M	38-year-old male	60-year-old male
Clinical Diagnosis & History	GERD, colon polyps	Colon polyps	Pan-colonic ulcerative colitis (UC), mild diverticulosis in the colon
Location of Tumor	Rectal polyp (~8 cm from anal verge), < 1 cm	Rectal lesion (~1.5 mm)	Rectal nodule (~7 cm from anal verge), < 1 cm
Staging	T1N0M0 (Stage I)	T1N0M0 (Stage I)	T1N0M0 (Stage I)
Histopathological Features	G1 NET; submucosal prolif.; Ki-67 ~2%; no lymphovascular invasion	G1 NET; superficial submucosal invasion; Ki-67 2.6%; no lymphovascular invasion	G1 NET; submucosal extension; Ki-67 <2%; no lymphovascular invasion
IHC Markers	SYN+ CHR- LU5+ CDX2+	SYN+ CHR- LU5+ CDX2 (Not Performed)	SYN+ CHR+ LU5 (Not Performed) CDX2 (not performed)

## Discussion

This case series highlights several key clinical characteristics of small rectal NETs that were identified by chance. All three patients had lesions with the largest dimension of less than 1 cm. They were thus categorized as Grade 1 based on their mitotic rate and Ki-67 index (≤3% in each case). Each tumor exhibited a characteristic neuroendocrine morphology and lacked significant atypia or high-risk histologic features. All patients were managed with endoscopic excision with no complications. These outcomes are consistent with multiple guidelines that recommend conservative therapy for well-differentiated, subcentimeter rectal NETs without invasive features [5,12-16].

In all three cases, the tumors were confined to the submucosa, showed Grade 1 morphology with a low Ki-67 index, and lacked lymphovascular or perineural invasion. These features correspond to a low-risk category in contemporary ENETS- and NCCN-informed frameworks for rectal NETs ≤10 mm, for which local endoscopic resection and surveillance are generally recommended over radical surgery. The fact that all lesions in our series were incidentally detected during evaluations for other indications is also consistent with the notion that many screen-detected, subcentimeter rectal NETs follow an indolent clinical course.

The case of Patient B demonstrates the critical decision-making required when treating small rectal NETs with narrow margins. Even though the lesion was close to the resection margin (1.5 mm), a conservative approach was supported by several favorable characteristics, including a low Ki-67 index (2.6%), minimal mitotic activity, and no lymphovascular invasion or deeper tissue involvement. Further lowering the risk of recurrence included the tumor's well-differentiated nature, Grade 1 character, and absence of atypia. Current recommendations and literature suggest that observation may be justified in such low-risk situations [17]. During follow-up, Patient B remained stable and asymptomatic, confirming the appropriateness of surveillance in the absence of high-risk characteristics.

The case of Patient C is noteworthy in the context of inflammatory bowel illness. Several studies in patients with chronic UC have reported the presence of microcarcinomas and carcinoid tumors. These findings suggest that persistent mucosal inflammation in UC may contribute to the overgrowth of neuroendocrine cells and the formation of carcinoid tumors [6,9-11]. However, a causal association remains in question, as evident by some studies.

For instance, in a comprehensive study by Greenstein and colleagues, carcinoid tumors were claimed to be detected by chance [18]. Notably, in a study examining 21 microcarcinoid foci in a colectomy specimen from a patient with UC, the researchers suggested that there was likely a reaction to chronic inflammation rather than a neoplasm as the biopsies showed no dysplasia or tumors [10]. Nonetheless, the absence of dysplasia or active luminal inflammation in Patient C lends support to the idea that rectal NETs may develop independently of UC in some individuals.

Although each patient case offers clinical insight, further research should examine a larger patient sample size. Additionally, molecular and genetic studies were not performed in all cases, and this may have been beneficial. Lastly, rectal NETs of various sizes should be included, as our case series was limited to patients with tumors of subcentimeter size.

Our findings are consistent with previous research indicating that subcentimeter, well-differentiated rectal NETs without high-risk characteristics have a longer short-term prognosis following endoscopic excision. This emphasizes the importance of endoscopists and pathologists recognizing and thoroughly assessing even minor, incidental lesions; careful histopathologic assessment can guide safe, conservative therapy. Multidisciplinary communication between gastrointestinal and pathology teams is critical for identifying patients who can be safely monitored with surveillance. Overall, more long-term studies are needed to better define surveillance intervals, guide treatment with fewer side effects, and explore molecular or biomarker risk factors.

## Conclusions

Rectal NETs can be detected early during regular or symptom-based endoscopies, resulting in less invasive treatment and more favorable long-term outcomes. This case series highlights the value of early detection and histopathologic evaluation in directing treatment. Also, it demonstrates the safety and efficacy of endoscopic resection in the treatment of small well-differentiated rectal NETs. Careful post-resection monitoring is still essential, particularly in cases where the margins are not negative. Altogether, these three cases illustrate the favorable prognosis for patients with low-grade rectal NETs found incidentally and managed with a conservative, minimally invasive approach.
